# Defective recognition of LC3B by mutant SQSTM1/p62 implicates impairment of autophagy as a pathogenic mechanism in ALS-FTLD

**DOI:** 10.1080/15548627.2016.1170257

**Published:** 2016-05-09

**Authors:** Alice Goode, Kevin Butler, Jed Long, James Cavey, Daniel Scott, Barry Shaw, Jill Sollenberger, Christopher Gell, Terje Johansen, Neil J. Oldham, Mark S. Searle, Robert Layfield

**Affiliations:** a School of Life Sciences, University of Nottingham, Nottingham, UK; b School of Chemistry, University of Nottingham, Nottingham, UK; c Centre for Biomolecular Sciences, University of Nottingham, Nottingham, UK; d Molecular Cancer Research Group, Institute of Medical Biology, University of Tromsø - The Arctic University of Norway, Tromsø, Norway

**Keywords:** ALS, Atg8/LC3, autophagy, FTLD, LIR, SQSTM1/p62

## Abstract

Growing evidence implicates impairment of autophagy as a candidate pathogenic mechanism in the spectrum of neurodegenerative disorders which includes amyotrophic lateral sclerosis and frontotemporal lobar degeneration (ALS-FTLD). *SQSTM1*, which encodes the autophagy receptor SQSTM1/p62, is genetically associated with ALS-FTLD, although to date autophagy-relevant functional defects in disease-associated variants have not been described. A key protein-protein interaction in autophagy is the recognition of a lipid-anchored form of LC3 (LC3-II) within the phagophore membrane by SQSTM1, mediated through its LC3-interacting region (LIR), and notably some ALS-FTLD mutations map to this region. Here we show that although representing a conservative substitution and predicted to be benign, the ALS-associated L341V mutation of SQSTM1 is defective in recognition of LC3B. We place our observations on a firm quantitative footing by showing the L341V-mutant LIR is associated with a ∼3-fold reduction in LC3B binding affinity and using protein NMR we rationalize the structural basis for the effect. This functional deficit is realized in motor neuron-like cells, with the L341V mutant EGFP-mCherry-SQSTM1 less readily incorporated into acidic autophagic vesicles than the wild type. Our data supports a model in which the L341V mutation limits the critical step of SQSTM1 recruitment to the phagophore. The oligomeric nature of SQSTM1, which presents multiple LIRs to template growth of the phagophore, potentially gives rise to avidity effects which amplify the relatively modest impact of any single mutation on LC3B binding. Over the lifetime of a neuron, impaired autophagy could expose a vulnerability, which ultimately tips the balance from cell survival toward cell death.

## Introduction

ALS and FTLD are devastating and invariably fatal neurodegenerative diseases thought to belong to the same clinicopathological spectrum of disorders. Both sporadic and familial forms occur with more than 30 different genes linked to ALS-FTLD and showing different degrees of causality from major effects through to genetic predisposition. A number of different pathogenic mechanisms have been proposed with growing evidence suggesting that autophagy may be disturbed in ALS-FTLD; post mortem analyses of patient tissue reveal increased numbers of autophagosomes^1,2^ and several disease-associated proteins are regulators of autophagy. More specifically, mutations affecting the autophagy cargo receptors UBQLN2/ubiquilin-2, OPTN/optineurin and SQSTM1/p62 have been reported in ALS-FTLD families.^3^ Very recently mutations affecting the gene encoding TBK1 (TANK-binding kinase 1), a kinase which targets both OPTN and SQSTM1 thus linking the proteins in a common pathway, have been identified as a cause of familial ALS-FTLD.^4,5^ Autophagy is a critical pathway for the removal of damaged and aggregation-prone proteins and organelles; for example, in the ALS context mediating the turnover of aggregated forms of the major pathological protein TARDBP (TAR DNA binding protein),^6^ and is essential for neuronal survival.^7,8^

ALS-FTLD-associated mutants of the autophagy receptor OPTN are defective in the selective degradation of mitochondria via autophagy^9,10^ with a mechanism that may involve compromised maturation of autophagosomes.^11^ Further, depletion of *optn* in zebrafish is associated with a motor axonopathy, which is phenotypically similar to a model of ALS expressing mutant SOD1.^12^ Similarly, numerous missense and truncating mutations affecting the *SQSTM1* gene, which encodes the SQSTM1 protein, have over the past 4 years been identified in patients with ALS and FTLD.^13^ Curiously *SQSTM1* mutations are also found in patients with the skeletal disorder Paget disease of bone (PDB), including some variants that are common to both disease phenotypes^13^ and polymorphisms in other autophagy genes have recently been associated with PDB in a Spanish cohort.^14^ SQSTM1 is a multidomain scaffold protein involved in various signaling pathways and more recently characterized as a cargo receptor for ubiquitin-mediated autophagy.^15^ At least in osteoclasts derived from mice expressing the equivalent of a PDB and ALS-associated mutant SQSTM1^P392L^, markers indicative of dysregulation of autophagy are evident.^16^ In zebrafish, knockdown of *Sqstm1* is associated with a locomotor phenotype that can be rescued by the autophagy activator rapamycin, as well as wild-type human SQSTM1 but not the P392L mutant, suggesting that changes in SQSTM1-mediated autophagy are linked to the phenotype.^17^

ALS-FTLD-associated missense mutations of SQSTM1 are distributed in different functional domains throughout the primary sequence of the 440-residue protein^13^ and notably a subset of these mutations map to the LC3-interacting region (LIR) of the protein.^18^ The LIR is a critical motif that allows SQSTM1 to couple to the developing phagophore through the recognition of lipid-anchored LC3 proteins (mammalian family of orthologs of yeast Atg8), within the phagophore membrane. This highly conserved region is located between amino acids 321 to 342 of human SQSTM1^15,19^ with structural analyses indicating that the motif DDDWTHL (corresponding to residues 335 to 341) is directly involved in the interaction with LC3. One particular *SQSTM1* mutation, an L341V missense mutation identified in a Chinese patient with late-onset sporadic ALS,^18^ is of interest since it affects a residue within the LIR of SQSTM1. Indeed the crystal structure of an LIR peptide (residues 332 to 342) in complex with LC3B revealed that L341 forms direct contacts with LC3B within the surface binding cleft.^19^ Consistent with this observation an artificial mutation at the same position (L341A) in the 332 to 342 LIR peptide results in significantly reduced SQSTM1-LC3B complex in qualitative protein binding assays,^19^ as is also the case in a slightly longer LIR peptide (332 to 347).^20^ Here we show that the ALS-associated L341V mutation, although previously predicted to represent a benign substitution,^18^ actually exerts a quantifiable effect on LC3B binding in vitro and affects SQSTM1 recruitment into acidic autophagic vesicles in living cells. Our data supports a model in which certain *SQSTM1* mutations limit a critical step in autophagy and provides important functional evidence for a role of the autophagic pathway in ALS-FTLD.

## Results

### The L341V mutation affects the recognition of LC3B by SQSTM1

**Figure 1.**
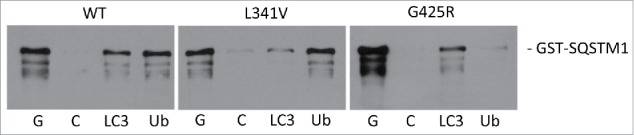
ALS-FTLD-associated SQSTM1 mutations impact on the recognition of LC3B (SQSTM1^L341V^) or ubiquitin (SQSTM1^G425R^) in vitro. Mutations as indicated (or wild type, WT) were introduced into the full-length GST-SQSTM1 sequence and affinity isolation assays (LC3B and ubiquitin on beads) were performed at 37°C. Bacterial lysates containing the GST-SQSTM1 fusions were incubated with glutathione- (G), control- (C), LC3B (LC3), and ubiquitin-Sepharose (Ub) beads and captured proteins were detected by western blotting (anti-SQSTM1 antibodies). A representative blot is shown; see Fig. S1 for quantification of 3 independent experiments.

The L341V missense mutation of *SQSTM1*, identified in a late-onset sporadic ALS patient, represents a conservative amino acid change, which has previously been predicted (based on SIFT and PolyPhen-2 prediction tools) to represent a benign substitution.^18^ We first used biochemical approaches to examine any impact of the L341V mutation on well-characterized protein-protein interactions relevant to SQSTM1-mediated autophagy. Protein affinity isolation assays in which interaction partners LC3B or ubiquitin covalently immobilized on beads were used to capture recombinant GST-SQSTM1 fusion proteins, followed by detection of bound protein by western blotting. The same assay has previously been used to demonstrate ubiquitin-binding defects associated with PDB-linked UBA domain mutations of SQSTM1.^21^ Consistent with its location within the SQSTM1 LIR, defined by the 338 to 341 WXXL motif, we found that the L341V mutant selectively reduced capture of GST-SQSTM1 by LC3B, relative to the wild type, whereas binding to ubiquitin was unaffected (Fig. 1 and Fig. S1). As a control the previously characterized PDB-linked UBA domain SQSTM1 mutation, G425R (SQSTM1^G425R^),^21^ also now reported in cases of ALS,^22^ was found to selectively impact only on ubiquitin-binding, with no evidence of this mutation affecting LC3B recognition. Thus, L341V exhibits a domain-specific effect on the interaction of SQSTM1 with LC3B in vitro.

### ESI-MS confirms weaker binding of the L341V mutant LIR to LC3B

**Figure 2.**
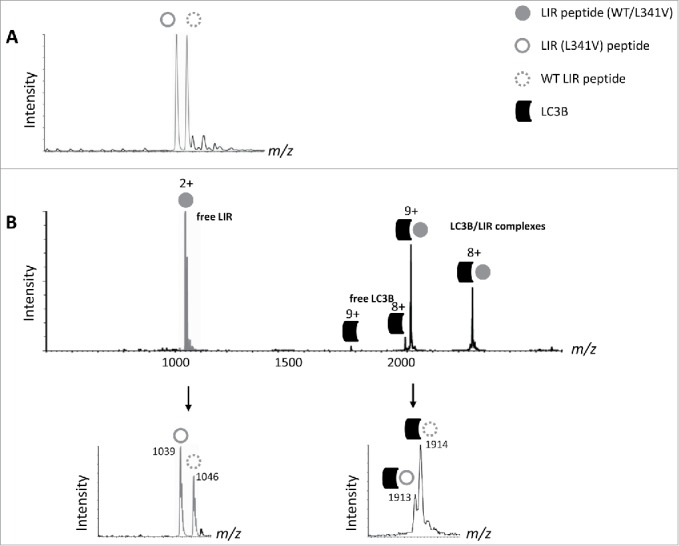
ESI-MS indicates weaker binding of the LIR (L341V) to LC3B compared to WT LIR. (A) Native ESI-MS spectrum of an equimolar mixture of WT LIR and LIR (L341V) peptides (5 µM, residues 332 to 351). (B) LIR peptide mixture titrated with 5 µM LC3B. Top, full spectrum indicating free LIR (gray filled circle, mixture of WT LIR and LIR [L341V]), free LC3B and LC3B-LIR complexes of indicated (multiple) charge states. Below, zoomed-in spectra showing *m/z* values of the free and bound LIR complexes at the indicated charge states.

We next used a native ESI-MS approach, where under carefully optimized nondenaturing conditions noncovalent protein complexes representative of solution binding can be maintained in the gas phase, in order to determine the effects of the L341V mutation on LC3B recognition by the LIR. Synthetic peptides with LIR (L341V) or without the mutation (WT LIR), representing an extended LIR sequence (residues 332 to 351) compared to those previously characterized in structural detail (332 to 342^19^ and 332 to 347^20^), were generated by solid-phase synthesis. Mass differences associated with the leucine to valine substitution meant that multiply charged species of the 2 peptides could be readily distinguished by ESI-MS (Fig. 2A). We set up a competitive binding mixture including 5 µM of purified LC3B along with equimolar concentrations of the 2 LIR peptides (i.e., a mixture of 1:1:1) and assessed complex formation by ESI-MS. Notably, peaks in the spectra showed evidence for a more significant population of the LC3B-WT LIR complex compared to LIR (L341V) (Fig. 2B, 2+ complex of free LIRs and 9+ complex of LIR-LC3B complexes indicated in lower panel) indicative of a reduction in binding affinity for the mutant peptide. Under these conditions the abundance ratio of LC3B bound to WT LIR compared to LIR (L341V) was 3:1, entirely consistent with a reduced binding affinity of the mutant LIR.

### Quantifying the magnitude of the effects of the L341V mutation on LC3B binding

**Figure 3.**
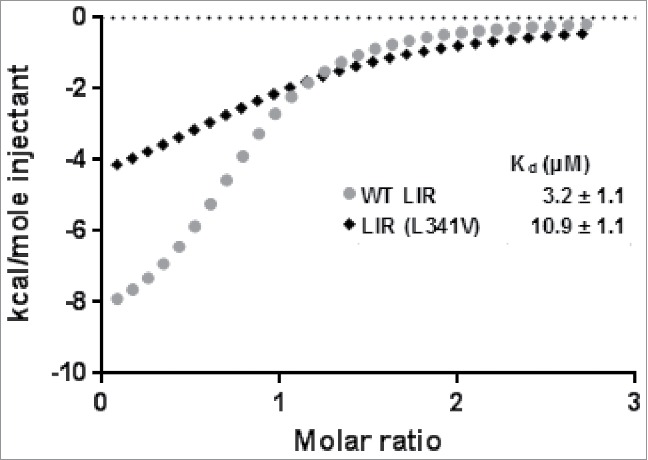
Isothermal titration calorimetry (ITC) binding isotherms from titrating LC3B with LIR peptides at 25°C. Binding isotherms were fitted to a one-site binding model and K_d_ values determined. Starting concentration of the LC3B was approximately 30 µM, and of LIR peptide (residues 332 to 351) stocks approximately 350 µM. Black diamond, LIR (L341V); gray circle WT LIR.

We used isothermal titration calorimetry (ITC) to corroborate the MS data and determine binding affinities (K_d_). Separate titrations were performed with starting concentration of the LC3B at 15 µM, with addition of aliquots of the LIR peptides (WT LIR or LIR (L341V) at 350 µM, at 25°C in 25 mM phosphate buffer, 150 mM NaCl, pH 7.5). High quality binding isotherms (Fig. 3) fitted well to a one-site binding model with a K_d_ value for WT LIR in the low μM range (3.2 ± 1.1 μM). However, the LIR (L341V) peptide was associated with a ∼3-fold reduction in LC3B binding affinity (10.9 ± 1.1 μM) and a substantially reduced enthalpy of interaction. The latter would appear to reflect possible steric effects and unfavorable van der Waals contacts from forcing a ‘square peg into a round hole.’ These binding affinities are within the same order of magnitude as previously published affinities from Novak et al.^23^ which use a similar-length peptide but covering a different set of residues around the LIR (residues 321 to 342).

### NMR reveals the structural basis for the effects of the L341V mutation on LC3B recognition

**Figure 4.**
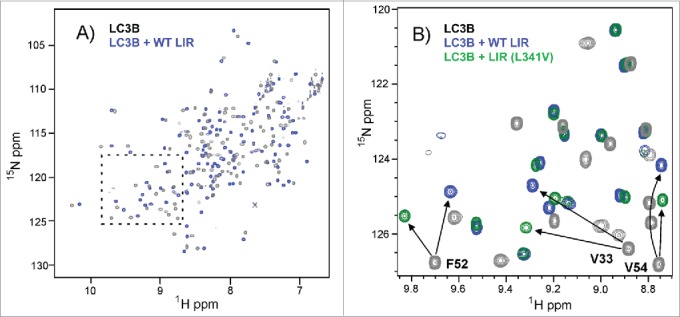
NMR titrations indicate selective perturbations of LC3B residues upon binding of LIR peptides. (A) ^1^H-^15^N-HSQC spectrum of LC3B (0.25 mM dark gray) overlaid with the spectrum of the complex of LC3B with WT LIR (0.5 mM blue, residues 332 to 351) (ratio of 1:2) showing extensive chemical shift perturbations (CSPs) across the spectrum induced by ligand binding at 298 K; (B) Expansion of the region highlighted in (A) showing overlap between the spectrum of LC3B (dark gray), LC3B+WT LIR (blue) and LC3B + LIR (L341V) (green) illustrating a number of key residues within the LIR binding pocket that are perturbed to different extents by the WT LIR and LIR (L341V). Arrows identify the shifts of V33, F52 and V54 in the 2 complexes, while the majority of other residues show only small differences between the spectra of the 2 complexes (overlap of blue and green peaks), demonstrating selective effects on residues in direct contact with the LIRs.

**Figure 5.**
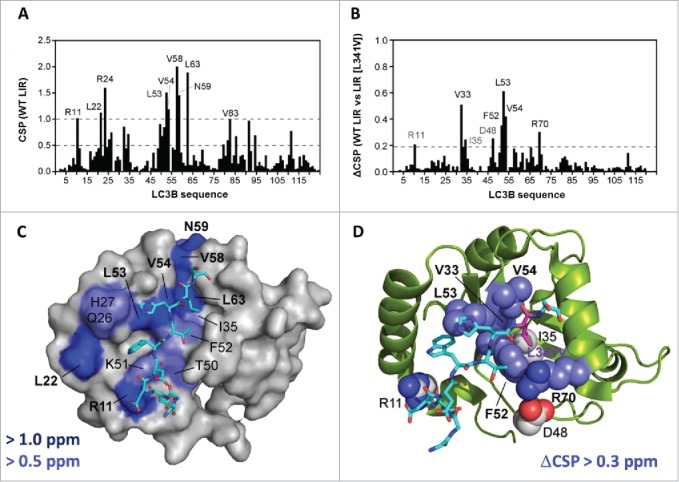
Chemical shift mapping of the binding of the WT LIR and LIR (L341V) peptides (residues 332 to 351) to ^15^N-LC3B. (A) Chemical shift mapping of the WT LIR. Weighted chemical shift pertubation (CSP) data showing the residues of ^15^N-LC3B (0.25 mM) that are perturbed upon WT LIR binding at a final ratio of 1:2 (LC3B:LIR) at 298 K. All CSPs above 1.0 are indicated. (B) Difference in CSP effects between WT LIR and LIR (L341V) sequences from NMR titrations at a final ratio of 1:2 (LC3B:LIR) at 298 K. The indicated residues showed CSPs that are substantially different between the 2 complexes (greater in the WT LIR). (C) Representation of binding surface of LC3B with WT LIR, generated by highlighting residues determined from NMR chemical shift mapping experiments. LC3B residues in blue showed the greatest CSP values. Backbone representation of the LIR peptide also indicated (residues DDDWTHLS, 335 to 342 shown). (D) Residues showing substantially different CSPs from the NMR titrations of LC3B with WT LIR compared to LIR (L341V), highlighted on the LC3B structure. Note the close correlation with the binding cleft in proximity to L341 of the LIR peptide (site of L341V indicated).

To understand the mechanism for impairment of binding of the LIR (L341V) at the molecular level we used NMR studies at 800 MHz to map differences in the binding interactions of LC3B, using the same WT LIR and LIR (L341V) peptide sequences used for MS and ITC studies. We first carried out an extensive amide backbone NMR assignment for LC3B (> 95% assignment of the nonprolyl residues) using double-isotope labeling with ^13^C/^15^N and a combination of 3D heteronuclear NMR spectra using well established methodologies (see Methods; Fig. S2, gray). Subsequently, we were able to use the sensitivity of ^1^H/^15^N-HSQC spectra of the assigned amide NHs at the individual residue level to map the perturbations to LC3B associated with ligand (LIR) binding (Fig. 4, Fig. S2, green and blue). Titration studies with WT LIR revealed significant changes to protein chemical shifts (> 0.5 ppm) for nearly 20 key residues; a subset of 9 of these demonstrated perturbations of > 1.0 ppm (Fig. 5A) indicative of substantial structural re-organization around the surface cleft to accommodate the bound ligand. These residues can be mapped to the surface of LC3B (Fig. 5C) and correspond very clearly with those identified as LIR contact residues in the X-ray structure of the LC3B-LIR (332 to 342) complex.^19^ Of particular note was the use of a shorter LIR-containing peptide in the X-ray studies (SGGDDDWTHLSS versus SGGDDDWTHLSSKEVDPSTG used here). Given that NMR CSP effects were observed over a substantial number of LC3B residues both in direct contact, and in some cases on the more remote nonbinding face, we compared NMR data with a truncated LIR peptide of only 11 residues (amino acids 335 to 345). The 2 titrations with the WT LIR-containing motifs highlighted a few small differences (data not shown), however, the largest perturbations were quantitatively very similar, indicating that the C-terminally extended peptide sequence used in our studies, which accommodates the additional VDPSTG motif corresponding to the KEAP1 interaction region (KIR), forms no additional significant interactions across the surface of LC3B.

We repeated the titration experiments with LIR (L341V) in the longer peptide sequence (332 to 351) and observed CSP effects of similar magnitude to WT LIR, showing the same extensive interaction (data not shown). By taking the difference in CSP effects between WT LIR and LIR (L341V) sequences we could readily identify 8 residues that were substantially different between the 2 complexes, with the largest effects involving V33, F52, L53, V54 and R70 (Fig. 5B). When these were specifically highlighted on the LC3B protein (Fig. 5D) they correlated remarkably closely with those constituting the binding cleft in proximity to L341. The perturbations demonstrate that binding of the mutant sequence is associated with some reorganization of the pocket to accommodate the different steric demands of the β-branched valine residue. We modeled the L341V substitution (data not shown), and observed significant steric clashes for all side chain rotamers of the substituted valine side chain, consistent with some loss of binding complementarity, leading to reduced affinity.

### Functional effects of the L341V mutation in a cellular context

To determine whether the L341V mutation affects autophagy in living cells we made use of an mCherry-EGFP double-tagged SQSTM1 construct which allows acidic autophagic vesicles to be distinguished from neutral SQSTM1-positive inclusion bodies and autophagosomes.^15^ When ectopically expressed in cells SQSTM1 can incorporate into cytoplasmic bodies with a similar phenotype to those observed when cells are stained for the endogenous protein.^24^ The inclusion bodies represent nonmembrane enclosed structures whereas the autophagosomes are membrane-limited; upon live cell imaging of both structures, red and green fluorescence (combined yellow) can be detected as each represent pH neutral microenvironments. However, following fusion of phagophores with lysosomes the EGFP (green) signal is quenched in the resulting acidic milieu, and only red fluorescence is detected, allowing transit of ectopically expressed SQSTM1 from phagophores to acidic autophagic vesicles to be monitored.

**Figure 6.**
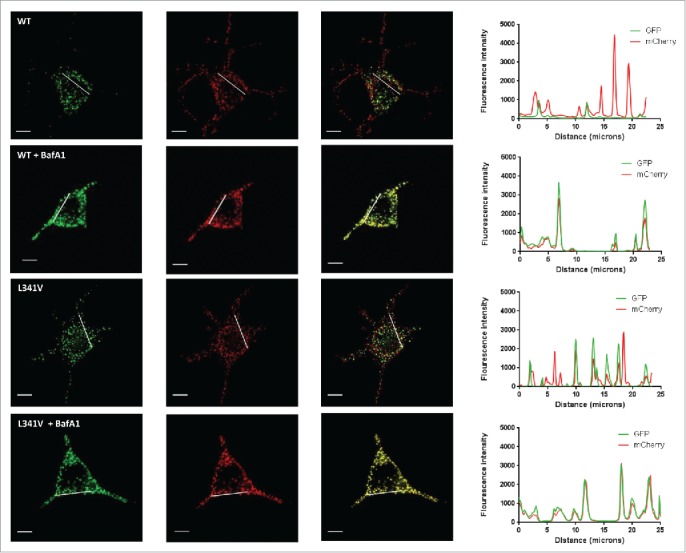
Imaging of NSC-34 cells transfected with mCherry-EGFP-SQSTM1 constructs. mCherry-EGFP-SQSTM1 constructs (wild type [WT] or L341V mutant) were transiently transfected into NSC-34 cells. Thirty-two h post-transfection cells were left untreated or treated with BafA1, 20 nM and 16 h later visualized on a DeltaVision microscope. Images shown are representative of cellular phenotypes of the vast majority of cells in each treatment group. Scale bars: 10 µm. (RHS) intensity plots along the lines as indicated on the extended focus cell images showing co-incidence of EGFP and mCherry fluorescence.

**Figure 7.**
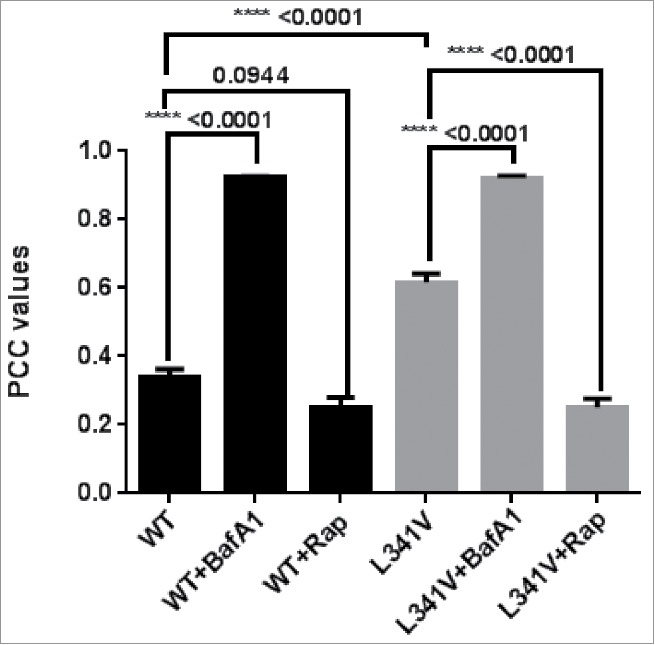
Quantification of colocalization of mCherry-EGFP-SQSTM1 transfected NSC-34 cells. Mean Pearson Correlation Coefficient (PCC) values of mCherry and EGFP overlap taken from a minimum of 50 cells per condition (NSC-34 cells transfected with mCherry-EGFP-SQSTM1 constructs) over a minimum of 3 independent experiments, with SEM indicated. Note the significant increase in PCC value in the wild-type-expressing cells treated with BafA1 and in the L341V mutant-expressing cells compared to wild type without BafA1 treatment (*P* values as indicated). When used cells were treated with 20 nM rapamycin 3 h before imaging (+Rap).

Previous studies demonstrate that deletion of the LIR strongly reduces the ability of SQSTM1 to be recruited into phagophores and subsequently acidic vesicles.^15^ To assess the impact of the more subtle L341V missense mutation, we introduced this change in to the mCherry-EGFP-SQSTM1 construct and transiently expressed this in the motor neuron-like cell line NSC-34. Notably endogenous levels of SQSTM1 are previously reported to be low (almost undetectable) in these cells.^25^ However, our more rigorous analysis using different SQSTM1 antibodies (Fig. S3) indicated that although NSC-34 cells do appear to express lower steady-state levels of SQSTM1 than other lines (adventitiously limiting the contribution of endogenous protein to any observed phenotype) these levels are not unusually low. Further, levels of endogenous SQSTM1 and LC3B increased in response to bafilomycin A_1_ (BafA1) treatment with a similar profile to HeLa cells (Fig. S3) indicating NSC-34 have normal autophagy machinery. We confirmed that steady-state levels of transfected mCherry-EGFP-SQSTM1 (wild type and L341V) were similar by western blot (Fig. S4). In cells 48 h after transfection with wild-type mCherry-EGFP-SQSTM1 we noted obvious yellow and red structures (Fig. 6),^26^ with the red acidic vesicles most prominent within the characteristic short neurites. Treatment with the autophagy inhibitor BafA1 visually reduced the number of red acidic structures (Fig. 6), which was quantified by determining the increase in the mean Pearson correlation coefficient (PCC) value of mCherry and EGFP overlap (Fig. 7). In contrast, in cells transfected with mutant mCherry-EGFP-SQSTM1^L341V^ there were visibly fewer red acidic vesicles in the absence of BafA1 (Fig. 6), evidenced by a mean PCC value which was greater than that for the wild type (Fig. 7). Treatment of the L341V mutant-expressing cells with BafA1 further increased the mean PCC value (Fig. 7), indicating that the cells were still functional with respect to autophagy, consistent with reversal of the mutant phenotype (reduction in PCC values) observed following treatment of mutant-expressing cells with the autophagy-enhancer rapamycin (Fig. 7). We also confirmed the L341V mutant phenotype in an additional neuronal cell line, mouse neuroblastoma Neuro-2a cells (Fig. S5); although the difference in mean PCC values between wild type- and L341V mutant-expressing cells was smaller in these cells this was still statistically significant. Overall these data are consistent with mutant mCherry-EGFP-SQSTM1^L341V^ being less readily incorporated into acidic vesicles than the wild type.

## Discussion

Our findings, using multiple independent approaches, of defective LC3B recognition by an ALS-associated variant of SQSTM1, and a reduced ability of the mutant protein to incorporate into acidic autophagic vesicles in living cells, adds to the accumulating evidence that disturbances in autophagy may underlie some cases of ALS-FTLD. Other studies to date have presented histological^1,2^ and genetic^3^ evidence for such a disturbance, but importantly we now add functional evidence by describing on a firm quantitative and structural footing a defect in an autophagy-relevant protein-protein interaction. Specifically we confirm that the ALS-associated L341V mutation of SQSTM1 exerts a quantifiable effect on LC3B binding in vitro. While a 3-fold reduction in binding affinity may appear modest, we suggest that over the lifetime of a neuron it may become biologically significant (see later). Indeed the change in binding affinity is sufficient to give a clear autophagy phenotype in our NSC-34 cell model as well as in Neuro-2a cells, i.e., two different neuronal cell lines, one of which is motor neuron-like. In this cellular context it is also possible that post-translational modification (e.g. phosphorylation of the LIR^27^) may directly or indirectly exaggerate the differences in binding affinities between wild-type and mutant sequences. However, a further important consideration in this respect is the structural organization of SQSTM1 in forming flexible oligomeric assemblies critical to its functional role in autophagy. Recent cryo-EM studies have established that the N-terminal (100 residue) PB1 domain of SQSTM1 and nearby sequences (also associated with ALS-related mutations) drive the formation of polymeric helical SQSTM1 filaments.^28^ These SQSTM1 filaments are proposed to present multiple LC3 binding sites to aid the growth of the phagophore onto the filaments. Thus, we speculate that avidity effects between multiple LIRs on the surface of the assembled SQSTM1 scaffold and membrane-tethered LC3B could provide a mechanism for amplifying any small detrimental effects on binding affinities from individual LIR mutations and these effects are ultimately manifested as a clear phenotype in the NSC-34 and Neuro-2a cell models. Similarly, in our in vitro affinity isolation with GST-SQSTM1, dimerization of GST may further promote avidity effects. Notably in the NSC-34 cells rapamycin is able to rescue the effects of the mutation on incorporation of SQSTM1 into phagophores, via currently undefined changes downstream of MTOR-dependent signaling, which must overcome the relatively modest reduction in LC3B-binding activity associated with the mutation; this observation supports the notion that autophagy-enhancing strategies may be beneficial in some cases of ALS.

The particular ALS-associated variant of SQSTM1 that we analyzed, identified in a late-onset sporadic ALS patient, is a conservative amino acid change (leucine to valine) which has previously been predicted by web-based prediction tools to represent a benign substitution.^18^ Such a prediction probably arose since L341 of SQSTM1, although highly conserved in mammals, in *Xenopus* is substituted by the valine amino acid found in the ALS patient. However it is not unusual for a deleterious missense mutation in one species to be found as the wild-type residue in orthologs of other species without effecting on the fitness of the latter; this can occur as a result of the phenomenon termed ‘Compensated Pathogenic Deviations'.^29^ Our functional studies are therefore a reminder of the caveats of relying on prediction programmes alone to establish the pathogenicity of variants of unknown clinical significance. It will be of particular importance in the future to extend our studies to other ALS-associated LIR mutations of SQSTM1 similarly suggested to be benign. For example, an aspartic acid to glutamic acid (D337E) mutation, also identified in a Chinese patient with sporadic ALS,^18^ is predicted to be benign. Although the artificial mutation of aspartic acid to alanine (D337A) reveals little effect on LC3B binding in affinity isolation assays using GST-tagged LIR sequences,^20^ subtle but biologically-relevant effects on LC3B recognition cannot be discounted. It is also notable that many ALS-associated SQSTM1 mutations affect other functional regions outside of the LIR that have significance for autophagy, for example the UBA domain and PB1 domains which are both necessary for successful cargo delivery to the phagophore.^3,15^ Therefore, while LIR mutations appear to limit the crucial step in selective autophagy of cargo receptor delivery to the phagophore, a model emerges where other SQSTM1 mutations may have an impact on, for example, cargo recognition, and the ability of the PB1 domain to regulate SQSTM1 assembly into the larger helical scaffolds required to nucleate the growing autophagosomal membrane.^28^ The role of avid interactions in amplifying the effects of individual mutations is intriguing in the light that SQSTM1 not only forms oligomeric scaffolds, but that the UBA domain also forms a dimeric structure that also plays a role in regulating binding to ubiquitinated cargo.^30^ In a similar context Lee et al. have recently highlighted how other ALS-associated genetic alterations may impact on different steps of autophagy, for example, vesicle nucleation (SOD1), autophagosome trafficking (DCTN [dynactin], DYNC1H1 [dynein cytoplasmic 1 heavy chain1], SOD1) and phagophore-lysosome fusion (VCP [valosin containing protein], CHMP2B [charged multivesicular body protein 2B]).^31^ Dysregulation of autophagy in ALS-FTLD may represent a wider mechanistic aberration that crosses over between other mutational and perhaps even nonmutational causes. The notion of autophagic dysfunction is entirely consistent with the emerging thinking that ‘lysosomal dysfunction’ underlies a range of neurodegenerative disorders including dementia with Lewy bodies and frontotemporal dementia (FTD, the most common FTLD syndrome), supported from recent genome-wide association study (GWAS) analyses.^32,33^

We speculate that subtle effects on autophagy associated with SQSTM1 (and other) variants predispose neurons to a vulnerability, which over time may become exposed by, for example, an age-related decline in autophagy pathway activity^34,35^ or ‘second-hit’ in another protein clearance pathway such as the ubiquitin-proteasome system.^6,36^ Causal mutations in other autophagy-related ALS-linked genes likely have more dominant effects. In this regard, Hardy and coworkers have recently speculated that motor neurons and cortical pyramidal neurons exist close to the edge of a ‘catastrophic cliff.’ Any events which expose neuronal vulnerability, for example mutations or other factors impacting on protein quality control, could be sufficient to push the cell ‘over the cliff’ i.e., ultimately resulting in cell death. In other words, subtle variants such as the L341V mutation of SQSTM1, over the lifetime of a neuron affect protein quality control, which in combination with other insults ultimately tip the balance from cell survival toward cell death. If the L341V mutant SQSTM1 were to accumulate over time (due to impaired turnover) then it would also have the potential to impact on the clearance of ubiquitinated proteins destined for proteasomal degradation by delaying their delivery to the proteasome.^37^ Presumably in such models, at the mechanistic level proteostasis defects ultimately drive the accumulation of proteins such as TARDBP in ALS and FTLD. If the model is found to be viable, therapies directed at clearing excess TARDBP would likely involve targeting a combination of these pathways.^38^

## Materials and methods

### Plasmids and peptides

The plasmids for expression of full-length wild-type and G425R mutant SQSTM1 protein (residues 1 to 440) as GST fusion proteins (pGEX-4T-1; GE Healthcare, 28-9545-49) in *E. coli* have been described previously.^21,39,40^ The L341V mutant construct was produced from the wild-type plasmid by site-directed mutagenesis (QuikChange II kit; Agilent Technologies, 200523) and subsequently verified by DNA sequencing. The L341V mutation was introduced into the wild-type pDEST-mCherry-EGFP-SQSTM1 construct^15^ via site-directed mutagenesis and verified by DNA sequencing. Custom peptides (residues 332 to 351 of human SQSTM1, SGGDDDWTHLSSKEVDPSTG (WT LIR) and SGGDDDWTHVSSKEVDPSTG (LIR [L341V]) were synthesized by PeptideSynthetics with a purity of > 98%. The cDNA of human *LC3B* was cloned into the pGEX-4T-3 plasmid (GE Healthcare, 28-9545-52) between the *Eco*RI and *SaI*I cloning sites. The cDNA of human *UBC* [ubiquitin C] was cloned into pKK233-3 (Promega, 27-4935-01) between *Eco*R1 and *Hind*III sites. Both the LC3B and ubiquitin were expressed by growing transformed BL21 (DE3) *E.coli* cells in either M9 minimal media or LB as described previously.^41^

### Protein purification

Ubiquitin was expressed and purified as described previously.^41^ The unlabeled, ^15^N-labeled and ^13^C/^15^N doubly-labeled LC3B was expressed as described previously for UBA domain constructs of SQSTM1.^30,41^ Thrombin cleavage was used to remove the GST-tag from glutathione-Sepharose-immobilized LC3B fusions. Purification of the eluted proteins using a salt gradient (0 to 2 M NaCl) on a cationic column (GE Healthcare, 17115201) in 5 mM potassium phosphate, pH 7, allowed fractions of purified LC3B to be concentrated by lyophilization. Fractions were then subsequently desalted on a 5 × 5 ml HiTrap^TM^ desalting column (GE Healthcare, 17-1408-01) in 25 mM ammonium acetate before further lyophilization.

### Ubiquitin and LC3B affinity isolation assays

Ubiquitin and LC3B affinity isolation assays of GST-SQSTM1 proteins were performed as described previously.^40^ Briefly, the GST fusion proteins were expressed in 10 ml cultures of *E. coli* and cells were lysed by sonicating in 1 ml TBS-T buffer (10 mM Tris, 150 mM NaCl, 0.1% v/v Triton-X100 [Thermo Scientific™, 85112], pH 7.5). The lysed cells were centrifuged at 16060 g for 10 min at room temperature and the cleared supernatant fractions were diluted 1:10 in TBS-T buffer. One ml of each diluted lysate was incubated at 37°C with excess glutathione-Sepharose (GE Healthcare, 17-0756-01), ubiquitin-Sepharose (10 mg/ml human ubiquitin immobilized on CNBr-activated Sepharose 4B [Sigma-Aldrich, C9142]), LC3B-Sepharose (1 mg/ml LC3B immobilized on CNBr-activated Sepharose 4B) or control-Sepharose (CNBr-activated Sepharose prepared without ubiquitin or LC3B). The unbound proteins were then removed and the beads washed with 3 × 1 ml TBS-T buffer at 37°C. Bound proteins were eluted from the beads with 50 μl of SDS PAGE loading buffer (10% 2-mercaptoethanol, 3% DTT, 20% glycerol, 2.5% SDS, 0.15 M Tris, 8 M urea, 0.1% bromophenol blue, pH 6.8). Bound proteins were revealed by western blotting with the mouse anti-SQSTM1 lck ligand antibody (BD Biosciences, 610833) which is insensitive to SQSTM1 UBA domain mutation status. Each assay was repeated on a minimum of 3 independent occasions and representative examples are presented. Immunoreactive bands were quantified by densitometry of 3 independent blots using Fiji (ImageJ); lanes were plotted and the area of intensity for each band quantified. The control-Sepharose values were subtracted from the glutathione-, LC3B- and ubiquitin-Sepharose values for each SQSTM1 sequence to account for nonspecific binding. LC3B- or ubiquitin-Sepharose values were divided by the respective glutathione-Sepharose values to normalize. Normalized values for the 2 GST-SQSTM1 mutants were then divided by the value for the wild type. GraphPad Prism 6 was used for statistical analysis in which a 2-way ANOVA with the Dunnett multiple comparisons test was performed to determine significance (significance set at 0.05).

### Electrospray ionization mass spectrometry (ESI-MS) competition assays

Native electrospray ionization mass spectrometry experiments (ESI-MS) described in this study, were performed using a Waters (Altrincham, UK) SYNAPT HDMS™, a hybrid quadrupole-ion mobility-orthogonal acceleration TOF instrument (oa-TOF). Three replicates of 6 titration samples were prepared as equal (v/v) solution mixtures in 25 mM ammonium acetate (pH 7) to the final concentrations of LC3B (5 μM) to each of the SQSTM1 LIR peptides (WT LIR and LIR [L341V]); 5 µM). The mixtures were directly infused into the mass spectrometer at a flow rate of 5 μL/min using a syringe pump (Harvard 22 dual syringe pump, model 55-2222 Holliston, MA, USA) and a 100 μL Hamilton syringe (Bonaduz, Switzerland). The mass spectrometer was operated in positive ion mode under the following optimized conditions: capillary voltage, 2.5 kV, cone voltage, 40 V, trap CE, 8 V, transfer CE, 5 V, backing pressure, ∼3.8 mbar, trap pressure, 2.1 × 10^−2^, TOF pressure. A quadrupole profile was applied which gave optimal ion transmission between 1000 to 4000 m/z. Spectra were acquired between 500 to 4000 m/z for 2 min. Instrument control as well as data processing was performed using MassLynx™ 4.1 software. Spectra were generated from the raw data acquired.

### Isothermal titration calorimetry (ITC)

Methods for isothermal titration calorimetry (ITC) have been described previously.^41,42^ In brief ITC was carried out using a MicroCal VP-ITC instrument. Ten µl of WT LIR or LIR (L341V) stock solution (350 µM) in 25 mM phosphate buffer, 150 mM NaCl, pH 7.5, was sequentially injected into the ITC cell containing 30 µM LC3B at 298 K in the same buffer solution, and the exothermic heat pulse was measured. Data were corrected for the effects of buffer mixing by including an enthalpy of mixing as an iterated variable. The data were analyzed using the MicroCal Origin software to determine a K_d_ using a global fit standard nonlinear least-squares regression analysis.

### Protein NMR experiments

3D NMR backbones assignments of LC3B (^1^H, ^13^Cα, ^13^Cβ and ^15^N for > 95% of non-prolyl residues) were carried out on Bruker AV(III)800 (Bruker UK Limited, Coventry, UK) and Bruker AV(III)600 NMR (Bruker UK Limited, Coventry, UK) spectrometers with a QCI cryoprobe and TXI room temperature probes respectively. Standard Bruker pulse sequences with Watergate solvent suppression at 298 K were used on a ^13^C, ^15^N isotopically labeled LC3B sample of 1 mM concentration in NMR buffer solution consisting of 25 mM potassium phosphate buffer, 50 mM NaCl, 5% (v/v) D_2_O (Sigma-Aldrich®, 151882), 0.02% (w/v) sodium azide, pH 7.0. The assignment was achieved using a combination of 2D and 3D experiments (^15^N-HSQC, HNCO, HN(CA)CO, HNCA, HN(CO)CA, HNCACB and HN(CO)CACB) using established assignment procedures.

NMR titration experiments were executed solely on the Bruker AV(III)800 NMR spectrometer using a QCI cryoprobe and standard pulse sequences with watergate solvent suppression. NMR titration studies of ^15^N-labeled LC3B (0.25 mM) with unlabeled LIR-peptides (WT LIR or LIR [L341V]) were performed at 298 K and ^1^H-^15^N-HSQC spectra were collected up to a LIR:LC3B ratio of 2:1 (i.e. up to 0.5 mM LIR). All NMR data was processed with TopSpin 3.1 (Bruker) and analysis and assignment was carried out in CcpNmr analysis 2.1.5 software.^43^ Chemical shift perturbations were calculated as Δδ_HSQC_ = [(Δδ_H_)^2^ + (Δδ_N_/5)^2^]^1/2^,^44^ where Δδ_H_ and Δδ_N_ are the observed shift perturbations in the ^1^H and ^15^N dimension of the HSQC spectrum.

### Fluorescence microscopy analyses

For live cell imaging the motor neuron-like cell line NSC-34 (a kind gift from Dr. Neil Cashman, University of Sheffield), a hybrid line produced by fusion of neuroblastoma with mouse motor neuron-enriched primary spinal cord cells,^45^ and the mouse neuroblastoma cell line Neuro-2a (a kind gift from Dr. Lynn Bedford, University of Nottingham) were used. Cells were cultured in Dulbecco's modified Eagle's medium (Sigma-Aldrich, D6429) supplemented with 1% (v/v) penicillin/streptomycin (Sigma-Aldrich, P0781) and 10% (v/v) FBS (Sigma-Aldrich, F9665) and kept in humidified 37°C, 5% CO_2_ incubators. Undifferentiated NSC-34 or Neuro-2a cells grown in glass bottom dishes (WillCo) were transfected using Lipofectamine 3000 (Invitrogen™, L3000-08) according to manufacturer's instructions with pDEST-mCherry-EGFP-SQSTM1 constructs (wild type or the L341V mutant). Thirty-two h after transfection cells were treated with +/−20 nM BafA1 (Calbiochem, 196000). Sixteen h later the media was removed from the cells, the cells were washed in PBS (Sigma-Aldrich, D8537) and then placed in Ringer buffer (2 mM CaCl_2_, 10 mM glucose, 10 mM HEPES, 5 mM KCl, 2 mM MgCl_2_·6H_2_O, 155 mM NaCl, 2 mM NaH_2_PO_4_·H_2_O, pH 7.2) and directly examined by wide-field fluorescence microscopy at 37°C for up to one h. Cells undergoing rapamycin treatment were treated as for cells without BafA1 but with 20 nM rapamycin (VIVA Bioscience Ltd., VB2112) added 3 h prior to imaging. Z-stacks were collected (200 nm spacing) using a DeltaVision Elite microscopy system (GE Healthcare Life Sciences, Buckinghamshire, UK) with a x60, 1.42 numerical aperture objective (Olympus, Southend-on-Sea, UK), images were recorded on a Photometric CoolSnap camera (Photometrics, Arizona, USA). The live cell filter wheel was used with the Quad-mCh polychroic. EGFP fluorescence was excited at 475 nm (bandwidth 28 nm) and the emission was recorded at 525 nm (bandwidth 25 nm). mCherry fluorescence was excited at 575 nm (bandwidth 25 nm) and the emission recorded at 632 nm (bandwidth 60 nm). Images of GFP and mCherry fluorescence were acquired sequentially for each z-stack slice. Single-cell image stacks underwent iterative restoration in either Volocity (Perkin Elmer) or Huygens Professional (Scientific Volume Imaging). Volocity was used to determine a Pearson correlation coefficient (PCC) value for each cell using the Costes method for estimating thresholds.^46^ Images of each condition were taken on a minimum of 3 independent occasions and a minimum of 50 cells per condition were analyzed. For every cell analyzed the PCC value was individually determined and the mean PCC values were calculated for all cells in each condition. GraphPad Prism 6 was used to determine if the PCC values between different conditions were statistically different using a one-way ANOVA analysis with multiple comparisons test comparing the mean of each column with the mean of every other column to report a multiplicity adjusted *P* value for each comparison (significance set at 0.05).

### Western blotting for autophagy markers

HeLa and NSC-34 cells were seeded at a density of 2 × 10^5^ cells/well in 12-well plates. Twenty-four h later cells were treated with varying concentrations of BafA1 (0, 1, 10 and 20 nM). Sixteen h after treatment the media was removed from the cells, the cells were washed in PBS and then harvested in fresh PBS. Cells were then pelleted at 380 g for 5 min in a table-top centrifuge and the PBS was removed. Cells were lysed in 300 μL of RIPA buffer (150 mM NaCl, 1% IGEPAL® CA-630 [Sigma-Aldrich®, I8896], 0.5% sodium deoxycholate [Sigma-Aldrich®,D6750], 0.1% SDS [Fisher Scientific, BP166], 50 mM Tris, pH 8.0, including protease [Sigma-Aldrich, P8340] and phosphatase [Sigma-Aldrich, P5726] inhibitors [at a 1:1000 dilution]). A BCA assay (ThermoFisher Scientific, 23225) determined the total protein concentration of each lysate and then lysates were separated by SDS PAGE on a 5–20% acrylamide gels with 20 μg of total protein loaded per well. Gels were transferred overnight and autophagy marker proteins were revealed by western blotting with the mouse anti-SQSTM1 lck ligand antibody, rabbit anti-SQSTM1 antibody (Enzo Life Sciences, BML-PW9860) and rabbit anti-LC3B antibody (MBL International, PM036). The blots were also stained for ACTB/β-actin (Sigma-Aldrich, A1978) to confirm equal protein loading.

## Supplementary Material

Supplementary_Figure_Legends.docx

2015AUTO0608R2-s06.pptx

2015AUTO0608R2-s05.pptx

2015AUTO0608R2-s04.pptx

2015AUTO0608R2-s03.pptx

2015AUTO0608R2-s02.pptx

## Abbreviations

ALS: amyotrophic lateral sclerosis
MAP1LC3B/LC3B: microtubule-associated protein 1 light chain 3 β (a mammalian ortholog of yeast Atg8)
CSP: chemical shift perturbation
EGFP: enhanced green fluorescent protein
ESI-MS: electrospray ionization mass spectrometry
FTLD: frontotemporal lobar degeneration
HSQC: heteronuclear single quantum correlation
ITC: isothermal titration calorimetry
KIR: KEAP1 interacting region
LIR: LC3-interacting region
NMR: nuclear magnetic resonance
PDB: Paget disease of bone 1
PB1: phox and bem1 domain
PBS: phosphate-buffered saline
PCC: Pearson correlation coefficient
SOD1: superoxide dismutase 1, soluble
SQSTM1/p62: sequestosome 1
TARDBP/TDP-43: TAR DNA binding protein
UBA: ubiquitin-associated domain
WT: wild type
