# Supplementary material for: Defective recognition of LC3B by mutant SQSTM1/p62 implicates impairment of autophagy as a pathogenic mechanism in ALS-FTLD
Source: Autophagy. 2016 May 9;12(7):1094–104. doi: 10.1080/15548627.2016.1170257 (PMC4990988; doi:10.1080/15548627.2016.1170257)
Supplement: Supplementary_Figure_Legends.docx [file kaup-12-07-1170257-s001.docx]

**Supplementary Figures**

<fig id="fig8"><number>**Figure S1.** Densitometric analysis (see Methods for details) showing quantification of representative western blot in <link rid="fig1">Fig. 1</link> combined with data from 2 other independent replicates; note reduced LC3B binding associated with mutant GST-SQSTM1^L341V^ and reduced ubiquitin-binding with the SQSTM1^G425R^ mutant, relative to wild type.</number></fig>

<fig id="fig9"><number>**Figure S2.** Overlaid ^1^H–^15^N-HSQC spectra from titrating LC3B (0.25 mM) with unlabelled WT LIR (blue, residues 332 to 351) or LIR (L341V) (green) at 298 K (0. 5 mM, ratio of 1:2).</number></fig>

<fig id="fig10"><number>**Figure S3.** NSC-34 or HeLa cells were treated with different concentrations of BafA1 for 16 h. Lysates were blotted for LC3B and SQSTM1 with samples normalized for total protein concentration (20 μg). Blotting for ACTB showed comparable protein levels between samples. Intensity of bands of LC3B-I, LC3B-II and SQSTM1 increased in a BafA1 dose-dependent manner. Note that although the mouse anti-SQSTM1 blot indicates that NSC-34 cells contain almost undetectable levels of endogenous SQSTM1, rabbit antibodies confirm expression of endogenous protein albeit at a lower level than in HeLa cells.</number></fig>

<fig id="fig11"><number>**Figure S4.** Western blot showing NSC-34 cell lysates (200 µg total protein) transfected with pDEST-mCherry-EGFP-SQSTM1 constructs as indicated and detected with anti-SQSTM1 (rabbit) and anti-ACTB antibodies, indicating comparable expression levels.</number></fig>

<fig id="fig12"><number>**Figure S5.** Mean Pearson Correlation Coefficient (PCC) values of mCherry and EGFP overlap taken from over 50 cells per condition (Neuro-2a cells transfected with mCherry-EGFP-SQSTM1 constructs) over 3 independent experiments, error bars representing SEM. Statistical significance is set at 0.05 with *P* values as indicated. Cells were treated with or without BafA1 as indicated.</number></fig>
