# Supplementary figures and images for: Defective recognition of LC3B by mutant SQSTM1/p62 implicates impairment of autophagy as a pathogenic mechanism in ALS-FTLD
Source: Autophagy. 2016 May 9;12(7):1094–104. doi: 10.1080/15548627.2016.1170257 (PMC4990988; doi:10.1080/15548627.2016.1170257)

## Slide 1
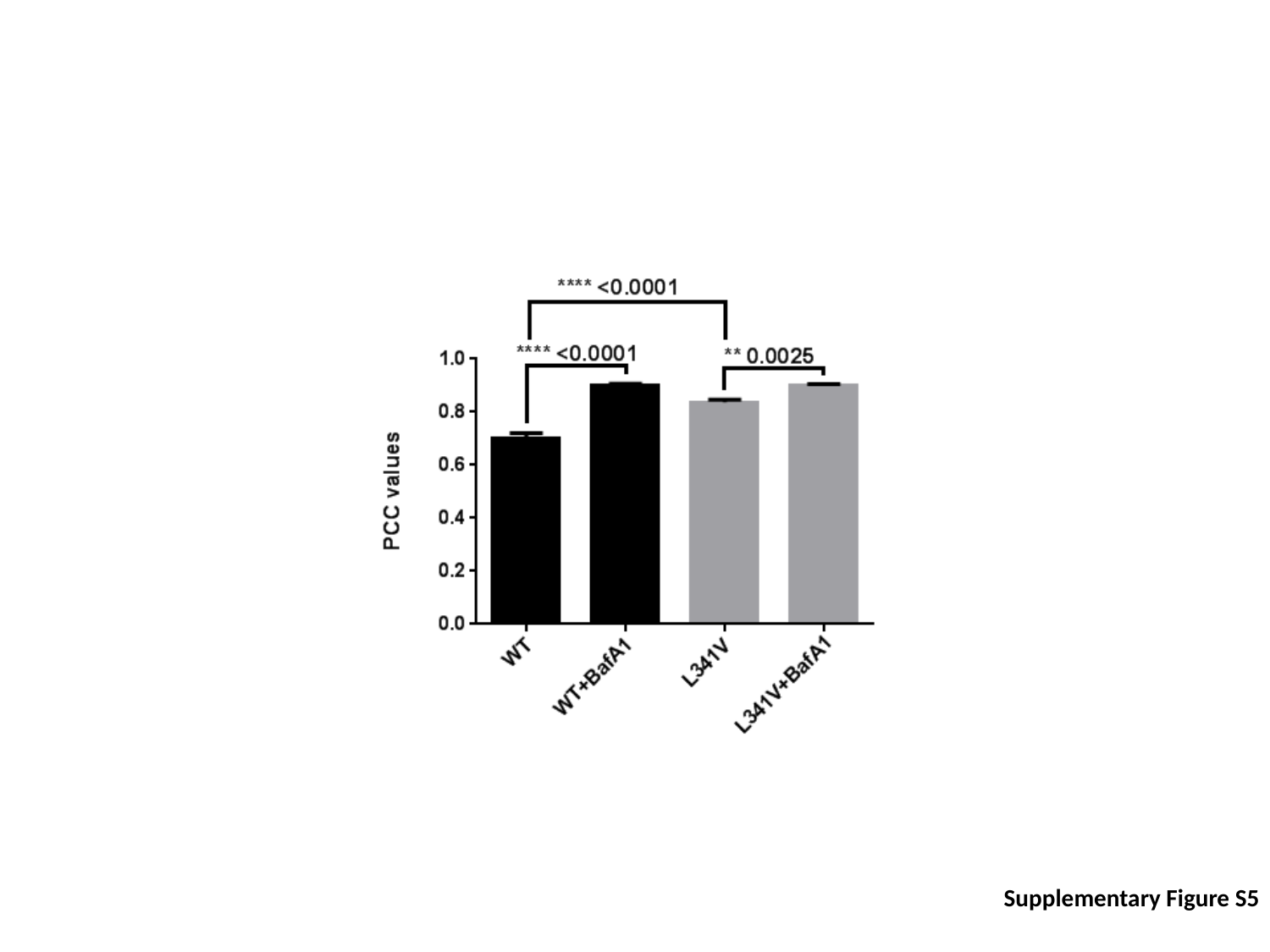

Supplementary Figure S5

Supplement: 2015AUTO0608R2-s06.pptx [file kaup-12-07-1170257-s002.pptx]

## Slide 1
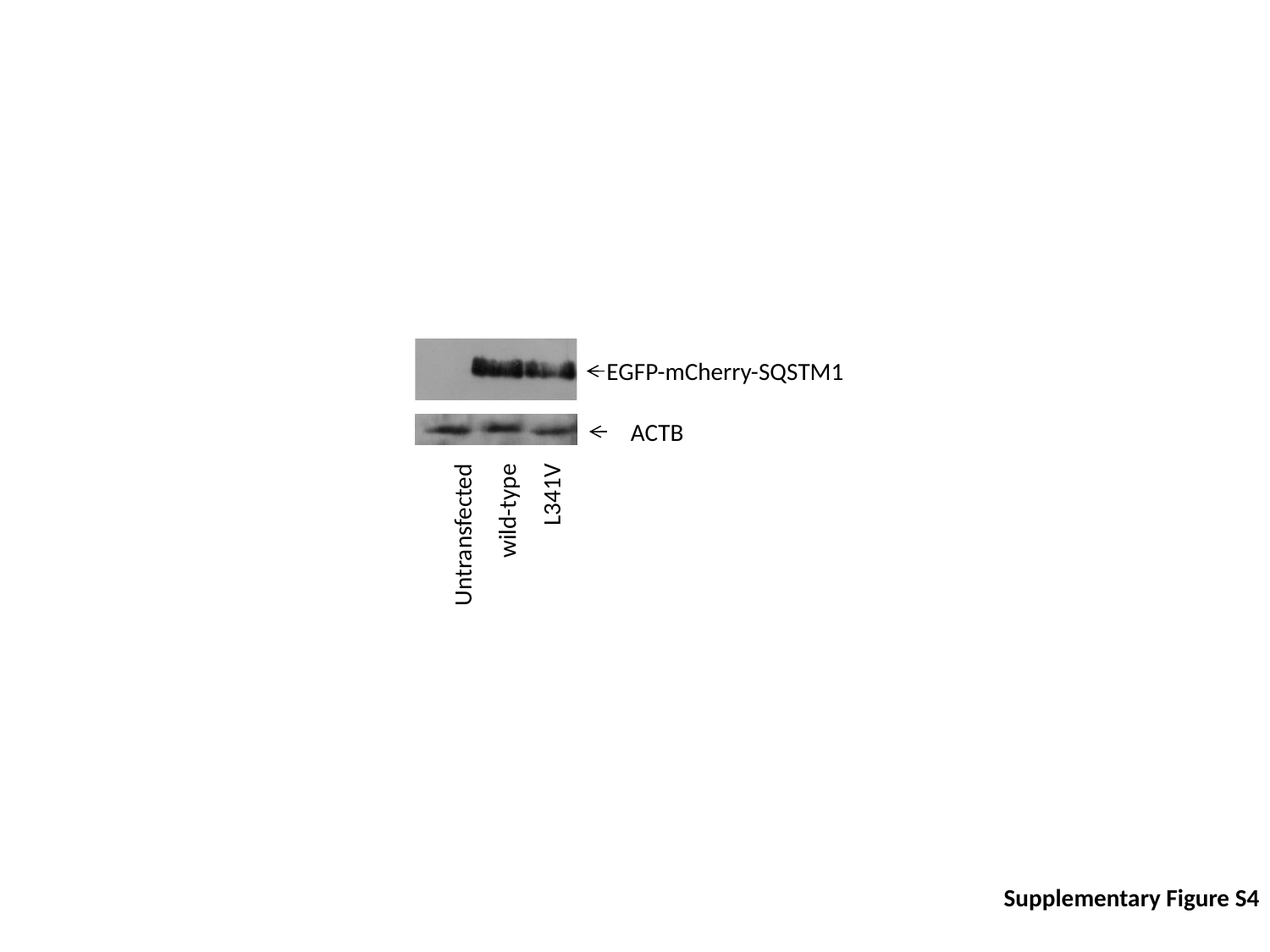

EGFP-mCherry-SQSTM1
ACTB
Untransfected
wild-type
L341V
Supplementary Figure S4

Supplement: 2015AUTO0608R2-s05.pptx [file kaup-12-07-1170257-s003.pptx]

## Slide 1
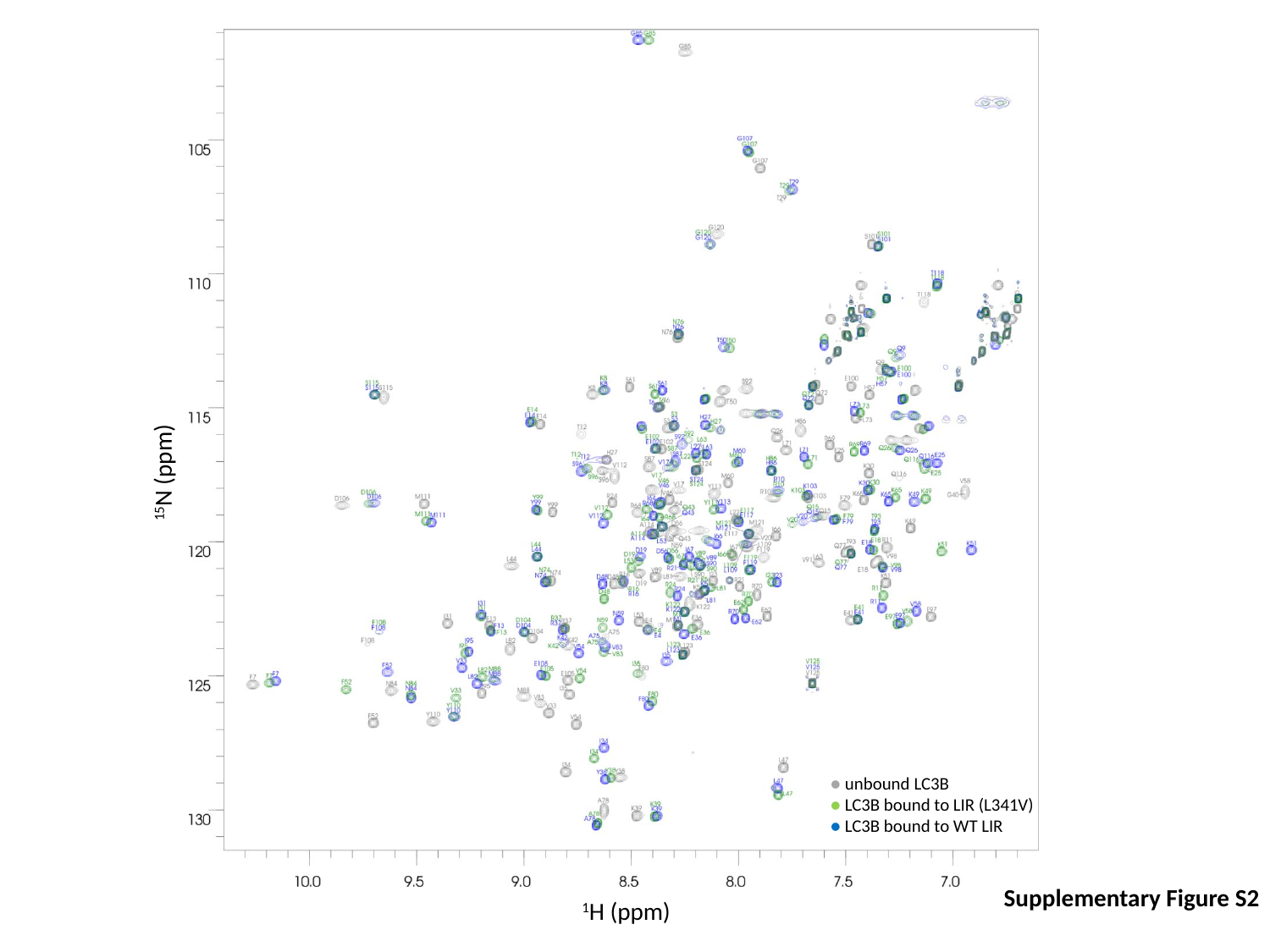

15N (ppm)
● unbound LC3B
● LC3B bound to LIR (L341V)
● LC3B bound to WT LIR
Supplementary Figure S2
1H (ppm)

Supplement: 2015AUTO0608R2-s03.pptx [file kaup-12-07-1170257-s005.pptx]

## Slide 1
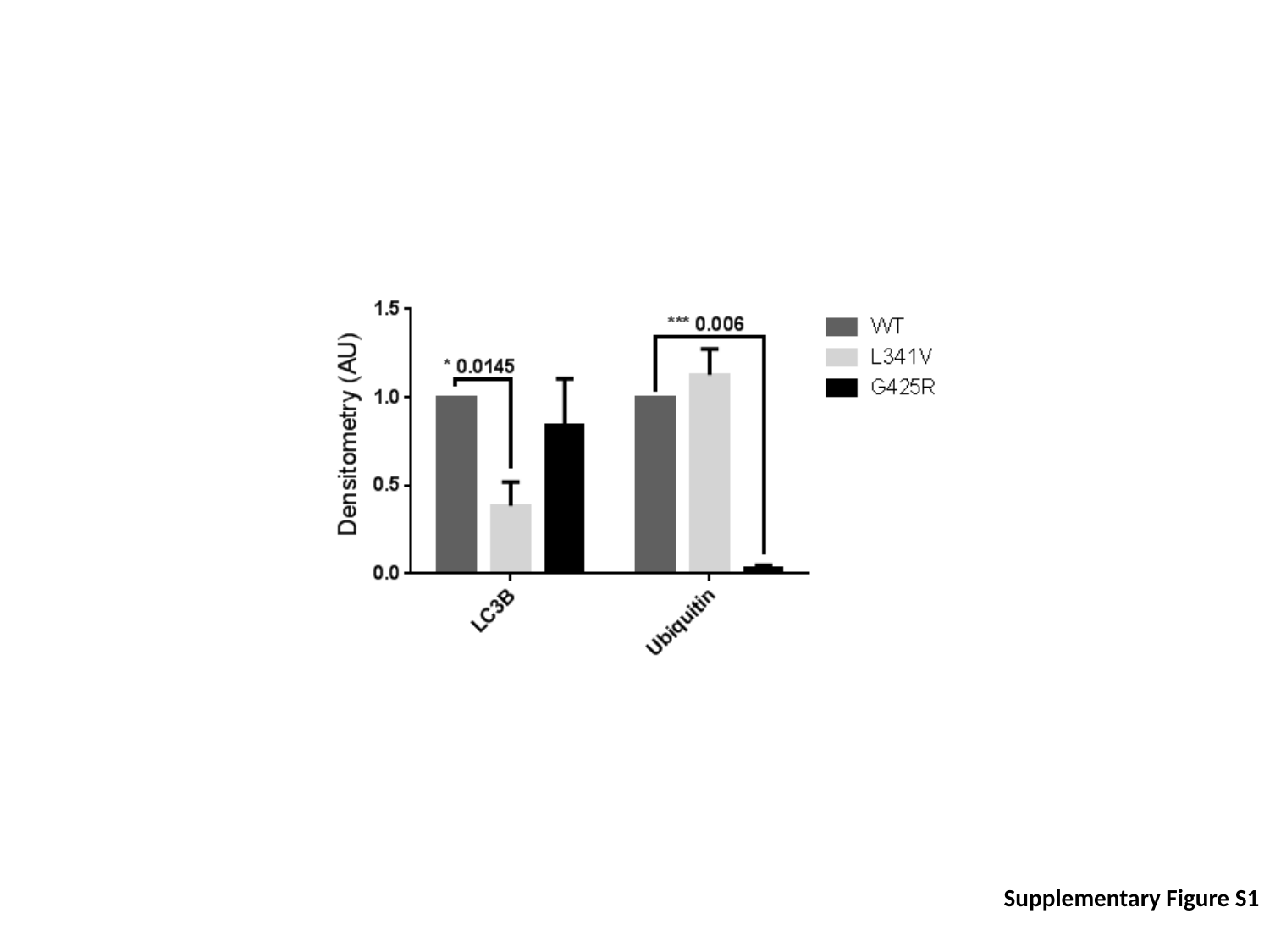

Supplementary Figure S1

Supplement: 2015AUTO0608R2-s02.pptx [file kaup-12-07-1170257-s006.pptx]
