# Supplementary material for: Defective recognition of LC3B by mutant SQSTM1/p62 implicates impairment of autophagy as a pathogenic mechanism in ALS-FTLD
Source: Autophagy. 2016 May 9;12(7):1094–104. doi: 10.1080/15548627.2016.1170257 (PMC4990988; doi:10.1080/15548627.2016.1170257)
Supplement: 2015AUTO0608R2-s04.pptx [file kaup-12-07-1170257-s004.pptx]

## Slide 1
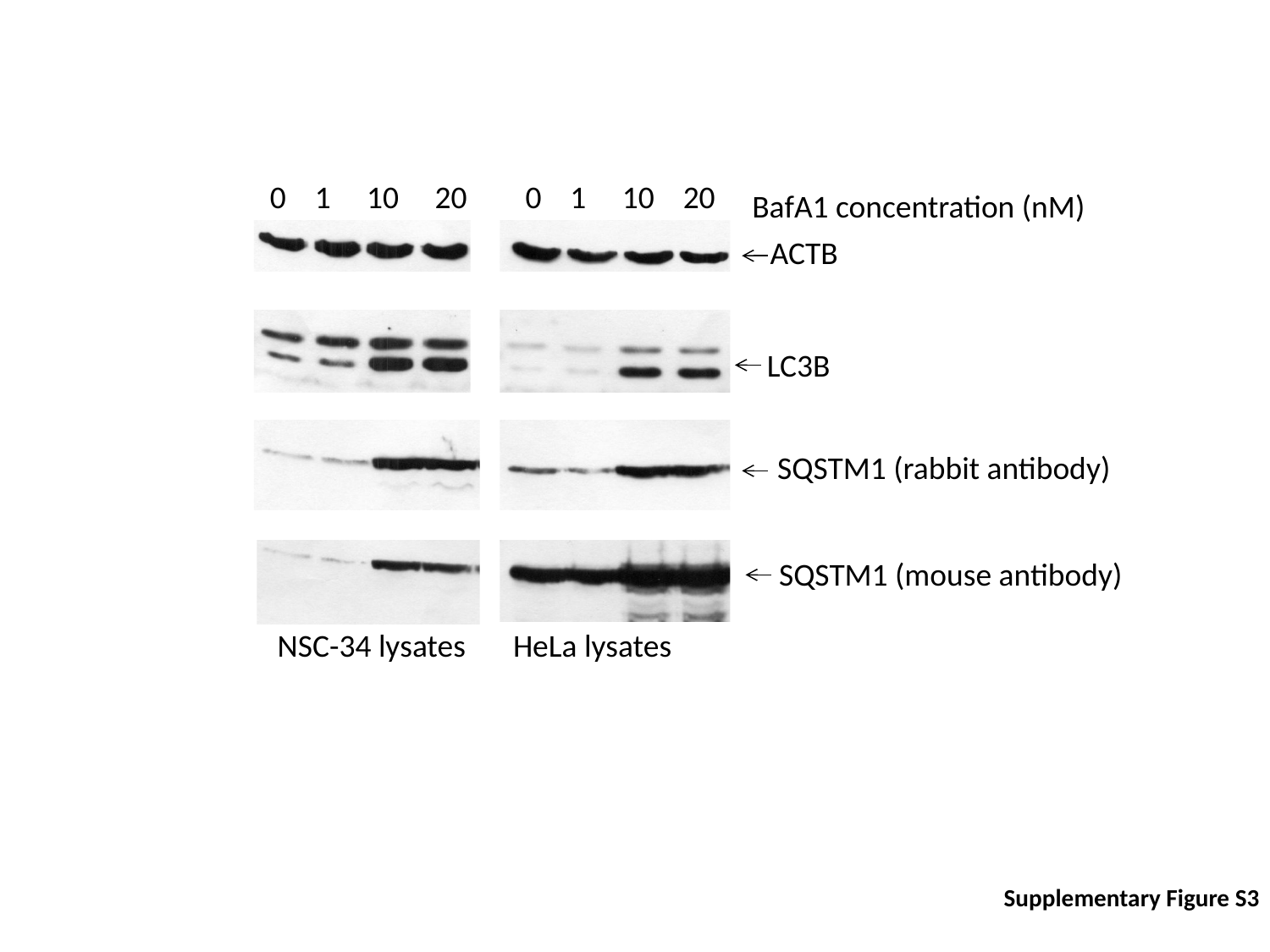

0 1 10 20
0 1 10 20
BafA1 concentration (nM)
ACTB
LC3B
SQSTM1 (rabbit antibody)
SQSTM1 (mouse antibody)
HeLa lysates
NSC-34 lysates
Supplementary Figure S3
